# Crystal structure of 1-ethyl­pyrazolo[3,4-*d*]pyrimidine-4(5*H*)-thione

**DOI:** 10.1107/S160053681401825X

**Published:** 2014-08-16

**Authors:** Mohammed El Fal, Youssef Ramli, El Mokhtar Essassi, Mohamed Saadi, Lahcen El Ammari

**Affiliations:** a Laboratoire de Chimie Organique Hétérocyclique URAC 21, Pôle de Compétence Pharmacochimie, Av. Ibn Battouta, BP 1014, Faculté des Sciences, Université Mohammed V-Agdal, Rabat, Morocco; b Laboratoire National de Contrôle des Médicaments, D M P, Ministère de la Santé, Madinat Al Irnane, BP 6206, Rabat, Morocco; c Laboratoire de Chimie du Solide Appliquée, Faculté des Sciences, Université Mohammed V-Agdal, Avenue Ibn Battouta, BP 1014, Rabat, Morocco

**Keywords:** crystal structure, pyrazolo­[3,4-*d*]pyrimidine, biological activity, hydrogen bonding

## Abstract

In the title compound, C_7_H_8_N_4_S, the methyl C atom is displaced by 1.232 (7) Å from the mean plane of the pyrazolo­[3,4-*d*]pyrimidine ring system (r.m.s. deviation = 0.007 Å). The N—N—C—C_m_ (m = meth­yl) torsion angle is −60.3 (6)°. In the crystal, mol­ecules are linked by N—H⋯S hydrogen bonds, generating [010] chains, which are reinforced by C—H⋯N inter­actions. The chains are cross-linked by weak C—H⋯S hydrogen bonds, generating (001) sheets.

## Related literature   

For the biological activity of pyrazolo­[3,4-*d*]pyrimidine deriv­atives, see: Rashad *et al.* (2008 ▶, 2011 ▶); Ballell *et al.* (2007 ▶). For related structures, see: El Fal *et al.* (2013 ▶); Radi *et al.* (2013 ▶); Alsubari *et al.* (2011 ▶).
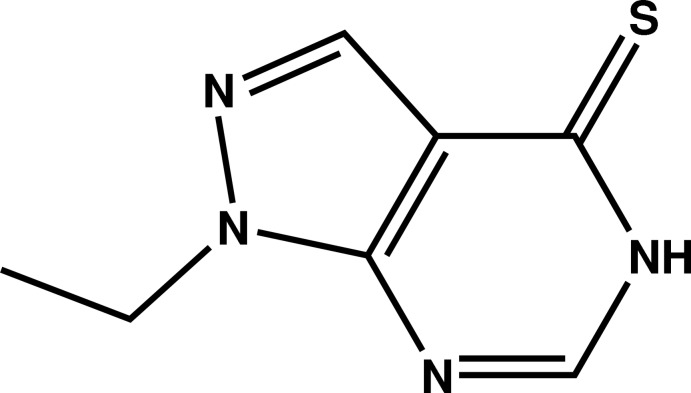


## Experimental   

### Crystal data  

C_7_H_8_N_4_S
*M* *_r_* = 180.23
Monoclinic, 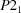

*a* = 4.472 (4) Å
*b* = 5.353 (4) Å
*c* = 17.573 (12) Å
β = 93.71 (4)°
*V* = 419.8 (5) Å^3^
*Z* = 2
Mo *K*α radiation
μ = 0.33 mm^−1^
*T* = 296 K
0.38 × 0.34 × 0.29 mm

### Data collection  

Bruker X8 APEX CCD diffractometer
Absorption correction: multi-scan (*SADABS*; Bruker, 2009 ▶) *T*_min_ = 0.578, *T*_max_ = 0.746
4028 measured reflections
1704 independent reflections
1242 reflections with *I* > 2σ(*I*)
*R*_int_ = 0.059

### Refinement  

*R*[*F* ^2^ > 2σ(*F* ^2^)] = 0.053
*wR*(*F* ^2^) = 0.139
*S* = 1.01
1704 reflections
109 parameters
1 restraint
H-atom parameters constrained
Δρ_max_ = 0.29 e Å^−3^
Δρ_min_ = −0.36 e Å^−3^
Absolute structure: Flack & Bernardinelli (2000 ▶), 652 Friedel pairs
Absolute structure parameter: −0.11 (16)

### 

Data collection: *APEX2* (Bruker, 2009 ▶); cell refinement: *SAINT-Plus* (Bruker, 2009 ▶); data reduction: *SAINT-Plus*; program(s) used to solve structure: *SHELXS97* (Sheldrick, 2008 ▶); program(s) used to refine structure: *SHELXL97* (Sheldrick, 2008 ▶); molecular graphics: *ORTEP-3 for Windows* (Farrugia, 2012 ▶); software used to prepare material for publication: *PLATON* (Spek, 2009 ▶) and *publCIF* (Westrip, 2010 ▶).

## Supplementary Material

Crystal structure: contains datablock(s) I. DOI: 10.1107/S160053681401825X/hb7262sup1.cif

Structure factors: contains datablock(s) I. DOI: 10.1107/S160053681401825X/hb7262Isup2.hkl

Supporting information file. DOI: 10.1107/S160053681401825X/hb7262Isup3.cml

**Fig. 1.**
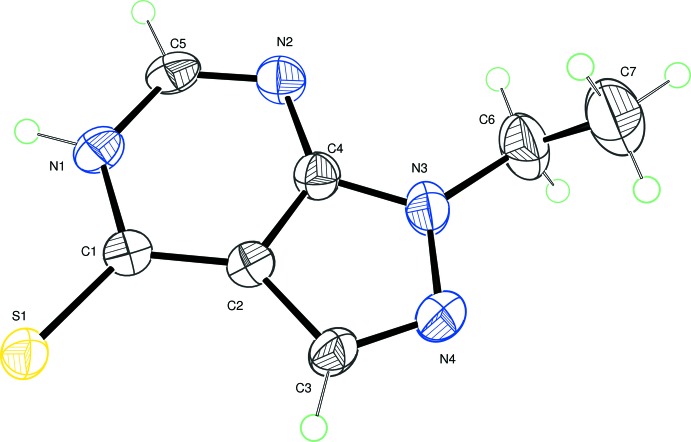
Molecular structure of the title compound with displacement ellipsoids drawn at the 50% probability level.

. DOI: 10.1107/S160053681401825X/hb7262fig1.tif
Mol­ecular structure of the title compound with displacement ellipsoids drawn at the 50% probability level.

**Fig. 2.**
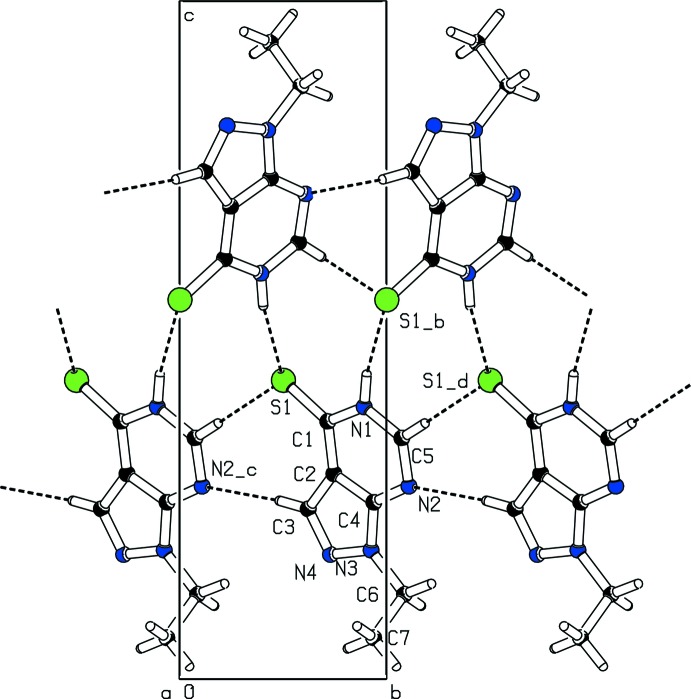
Structure projection along (0 1 1) of the title compound, showing molecules linked through hydrogen bonds (dashed lines).

. DOI: 10.1107/S160053681401825X/hb7262fig2.tif
Structure projection along (0 1 1) of the title compound, showing mol­ecules linked through hydrogen bonds (dashed lines).

CCDC reference: 1018657

Additional supporting information:  crystallographic information; 3D view; checkCIF report

## Figures and Tables

**Table 1:** Hydrogen-bond geometry (Å, °)

*D*—H⋯*A*	*D*—H	H⋯*A*	*D*⋯*A*	*D*—H⋯*A*
N1—H1⋯S1^i^	0.89	2.48	3.333 (4)	161
C5—H5⋯S1^ii^	0.93	2.75	3.685 (5)	179
C3—H3⋯N2^iii^	0.93	2.60	3.528 (6)	174

Symmetry codes: (i) 
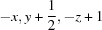
; (ii) 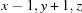; (iii) 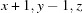.

## supplementary crystallographic information

### S1. Comment

Pyrazolo [3,4-*d*] pyrimidine derivatives have attracted considerable attention from researchers due to their bioactive and pharmaceutical properties. Many members of this family are widely used as antiviral (Rashad *et al.*, 2008); anti-mycobacterial (Ballell *et al.* 2007) and anticancer agents (Rashad *et al.* 2011). The present paper is a continuation of our research work devoted to the development of pyrazolo [3,4-*d*] pyrimidine derivatives with potential pharmacological activities (El Fal *et al.*, 2013; Radi *et al.*, 2013; Alsubari *et al.*, 2011).

The molecule of the title compound is build up from two fused five- and six-membered heterocycles linked to an ethyl group and to S atom as shown in Fig.1. The pyrazolo[3,4-*d*]pyrimidine ring is nearly perpendicular to the ethyl group as indicated by the torsion angle C7C6N3N4 of -60.3 (6)°.

In the crystal, the molecules are linked together by a weak intermolecular N1–H1···S1, C5–H5···S1 and C3–H3···N2 interactions, in the way to build a two-dimensional network (see Fig.2 and Table 1).

### S2. Experimental

(0,54 g, 3.04 mmol) of 1-ethyl-pyrazolo [3, 4 - d] pyrimidin-4 (5*H*)-one and (0,84 g, 3.65 mmol) of phosphorus pentasulfide were refluxed in pyridine for 4 h. Then the solvent is evaporated under reduced pressure; the precipitate formed is washed with hot water and recrystallized from ethanol solution to afford the title compound as yellow blocks.

### S3. Refinement

The H atoms were located in a difference map and treated as riding with C—H = 0.93 Å (aromatic), C—H = 0.97 Å (methylene) and C—H = 0.96 Å, (methyl). All hydrogen with *U*_iso_(H) = 1.2 *U*_eq_ (aromatic and methylene) and *U*_iso_(H) = 1.5 *U*_eq_ for the methyl.

### Crystal data

**Table:**

C_7_H_8_N_4_S	*F*(000) = 188
*M**_r_* = 180.23	*D*_x_ = 1.426 Mg m^−^^3^
Monoclinic, *P*2_1_	Mo *K*α radiation, λ = 0.71073 Å
Hall symbol: P 2yb	Cell parameters from 1704 reflections
*a* = 4.472 (4) Å	θ = 3.5–27.5°
*b* = 5.353 (4) Å	µ = 0.33 mm^−^^1^
*c* = 17.573 (12) Å	*T* = 296 K
β = 93.71 (4)°	Block, yellow
*V* = 419.8 (5) Å^3^	0.38 × 0.34 × 0.29 mm
*Z* = 2	

### Data collection

**Table:**

Bruker X8 APEX CCD diffractometer	1704 independent reflections
Radiation source: fine-focus sealed tube	1242 reflections with *I* > 2σ(*I*)
Graphite monochromator	*R*_int_ = 0.059
φ and ω scans	θ_max_ = 27.5°, θ_min_ = 3.5°
Absorption correction: multi-scan (*SADABS*; Bruker, 2009)	*h* = −5→5
*T*_min_ = 0.578, *T*_max_ = 0.746	*k* = −6→5
4028 measured reflections	*l* = −22→22

### Refinement

**Table:**

Refinement on *F*^2^	Secondary atom site location: difference Fourier map
Least-squares matrix: full	Hydrogen site location: inferred from neighbouring sites
*R*[*F*^2^ > 2σ(*F*^2^)] = 0.053	H-atom parameters constrained
*wR*(*F*^2^) = 0.139	*w* = 1/[σ^2^(*F*_o_^2^) + (0.*P*)^2^] where *P* = (*F*_o_^2^ + 2*F*_c_^2^)/3
*S* = 1.01	(Δ/σ)_max_ < 0.001
1704 reflections	Δρ_max_ = 0.29 e Å^−^^3^
109 parameters	Δρ_min_ = −0.36 e Å^−^^3^
1 restraint	Absolute structure: Flack & Bernardinelli (2000), 652 Friedel pairs
Primary atom site location: structure-invariant direct methods	Absolute structure parameter: −0.11 (16)

### Special details

**Table:**

Geometry. All e.s.d.'s (except the e.s.d. in the dihedral angle between two l.s. planes) are estimated using the full covariance matrix. The cell e.s.d.'s are taken into account individually in the estimation of e.s.d.'s in distances, angles and torsion angles; correlations between e.s.d.'s in cell parameters are only used when they are defined by crystal symmetry. An approximate (isotropic) treatment of cell e.s.d.'s is used for estimating e.s.d.'s involving l.s. planes.
Refinement. Refinement of *F*^2^ against all reflections. The weighted *R*-factor *wR* and goodness of fit *S* are based on *F*^2^, conventional *R*-factors *R* are based on *F*, with *F* set to zero for negative *F*^2^. The threshold expression of *F*^2^ > σ(*F*^2^) is used only for calculating *R*-factors(gt) *etc*. and is not relevant to the choice of reflections for refinement. *R*-factors based on *F*^2^ are statistically about twice as large as those based on *F*, and *R*- factors based on all data will be even larger.

### Fractional atomic coordinates and isotropic or equivalent isotropic displacement parameters (Å^2^)

**Table:**

	*x*	*y*	*z*	*U*_iso_*/*U*_eq_	
C1	0.2656 (8)	0.2169 (7)	0.6200 (2)	0.0340 (9)	
C2	0.3666 (8)	0.2465 (8)	0.6972 (2)	0.0349 (9)	
C3	0.5729 (9)	0.1222 (9)	0.7490 (2)	0.0421 (10)	
H3	0.6856	−0.0166	0.7370	0.050*	
C4	0.2506 (9)	0.4396 (7)	0.7391 (2)	0.0386 (10)	
C5	−0.0448 (9)	0.5752 (8)	0.6444 (2)	0.0438 (11)	
H5	−0.1874	0.6853	0.6230	0.053*	
C6	0.3360 (13)	0.5872 (10)	0.8749 (3)	0.0669 (16)	
H6A	0.1925	0.7165	0.8595	0.080*	
H6B	0.5229	0.6675	0.8919	0.080*	
C7	0.2207 (15)	0.4383 (15)	0.9390 (3)	0.091 (3)	
H7A	0.1876	0.5471	0.9810	0.137*	
H7B	0.3652	0.3131	0.9550	0.137*	
H7C	0.0355	0.3592	0.9221	0.137*	
N1	0.0543 (7)	0.3928 (6)	0.59849 (19)	0.0407 (9)	
H1	−0.0256	0.4046	0.5509	0.049*	
N2	0.0419 (8)	0.6091 (7)	0.71546 (19)	0.0435 (9)	
N3	0.3849 (9)	0.4260 (7)	0.80986 (17)	0.0482 (10)	
N4	0.5830 (8)	0.2300 (8)	0.81616 (19)	0.0513 (10)	
S1	0.3799 (2)	0.0022 (2)	0.55937 (5)	0.0413 (3)	

### Atomic displacement parameters (Å^2^)

**Table:**

	*U*^11^	*U*^22^	*U*^33^	*U*^12^	*U*^13^	*U*^23^
C1	0.0340 (18)	0.030 (2)	0.038 (2)	−0.0022 (18)	0.0034 (16)	0.0046 (17)
C2	0.0340 (18)	0.030 (2)	0.040 (2)	−0.0013 (19)	0.0000 (15)	0.0005 (18)
C3	0.046 (2)	0.039 (2)	0.040 (2)	0.011 (2)	−0.0064 (17)	0.0030 (19)
C4	0.041 (2)	0.036 (3)	0.038 (2)	−0.0003 (19)	0.0021 (16)	−0.0011 (17)
C5	0.036 (2)	0.038 (3)	0.056 (3)	0.0102 (19)	−0.0030 (18)	0.005 (2)
C6	0.089 (4)	0.067 (4)	0.045 (3)	0.002 (3)	0.005 (2)	−0.017 (2)
C7	0.094 (4)	0.128 (8)	0.053 (3)	−0.003 (5)	0.016 (3)	−0.009 (3)
N1	0.0406 (18)	0.037 (2)	0.0440 (19)	0.0066 (17)	−0.0044 (15)	0.0078 (16)
N2	0.050 (2)	0.034 (2)	0.046 (2)	0.0076 (18)	0.0027 (16)	−0.0015 (16)
N3	0.059 (2)	0.052 (3)	0.0337 (17)	0.0086 (19)	0.0001 (15)	−0.0048 (16)
N4	0.051 (2)	0.058 (3)	0.044 (2)	0.015 (2)	−0.0072 (16)	−0.0002 (18)
S1	0.0449 (5)	0.0390 (6)	0.0388 (5)	0.0052 (6)	−0.0053 (4)	−0.0052 (5)

### Geometric parameters (Å, º)

**Table:**

C1—N1	1.370 (5)	C5—H5	0.9300
C1—C2	1.410 (5)	C6—N3	1.459 (5)
C1—S1	1.669 (4)	C6—C7	1.499 (7)
C2—C4	1.390 (5)	C6—H6A	0.9700
C2—C3	1.419 (6)	C6—H6B	0.9700
C3—N4	1.312 (5)	C7—H7A	0.9600
C3—H3	0.9300	C7—H7B	0.9600
C4—N3	1.347 (5)	C7—H7C	0.9600
C4—N2	1.348 (5)	N1—H1	0.8900
C5—N2	1.296 (5)	N3—N4	1.373 (5)
C5—N1	1.359 (5)		
			
N1—C1—C2	111.1 (3)	N3—C6—H6B	109.5
N1—C1—S1	122.1 (3)	C7—C6—H6B	109.5
C2—C1—S1	126.8 (3)	H6A—C6—H6B	108.1
C4—C2—C1	119.1 (4)	C6—C7—H7A	109.5
C4—C2—C3	104.9 (3)	C6—C7—H7B	109.5
C1—C2—C3	136.0 (4)	H7A—C7—H7B	109.5
N4—C3—C2	110.8 (4)	C6—C7—H7C	109.5
N4—C3—H3	124.6	H7A—C7—H7C	109.5
C2—C3—H3	124.6	H7B—C7—H7C	109.5
N3—C4—N2	125.4 (4)	C5—N1—C1	125.2 (3)
N3—C4—C2	106.9 (4)	C5—N1—H1	112.4
N2—C4—C2	127.7 (3)	C1—N1—H1	122.4
N2—C5—N1	125.7 (4)	C5—N2—C4	111.2 (3)
N2—C5—H5	117.1	C4—N3—N4	111.2 (3)
N1—C5—H5	117.1	C4—N3—C6	127.6 (4)
N3—C6—C7	110.5 (5)	N4—N3—C6	121.2 (4)
N3—C6—H6A	109.5	C3—N4—N3	106.2 (3)
C7—C6—H6A	109.5		

### Hydrogen-bond geometry (Å, º)

**Table:**

*D*—H···*A*	*D*—H	H···*A*	*D*···*A*	*D*—H···*A*
N1—H1···S1^i^	0.89	2.48	3.333 (4)	161
C5—H5···S1^ii^	0.93	2.75	3.685 (5)	179
C3—H3···N2^iii^	0.93	2.60	3.528 (6)	174

Symmetry codes: (i) −*x*, *y*+1/2, −*z*+1; (ii) *x*−1, *y*+1, *z*; (iii) *x*+1, *y*−1, *z*.
