# Supplementary figures and images for: Crystal structure of 1-ethyl­pyrazolo[3,4-d]pyrimidine-4(5H)-thione
Source: Acta Crystallogr Sect E Struct Rep Online. 2014 Aug 16;70(Pt 9):o1005–6. doi: 10.1107/S160053681401825X (PMC4186190; doi:10.1107/S160053681401825X)

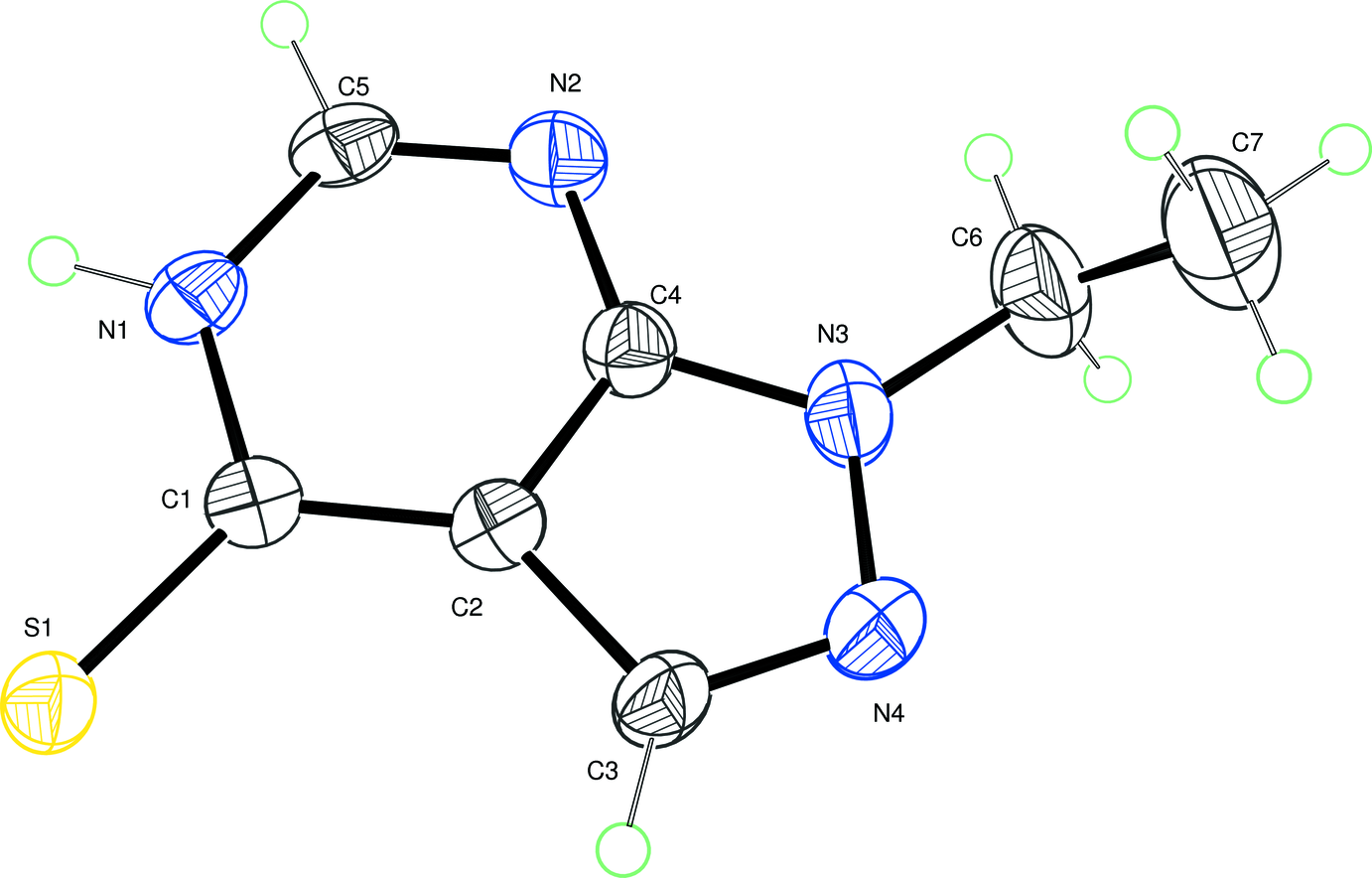

Supplement: Supplementary file 4 [file e-70-o1005-fig1.tif]

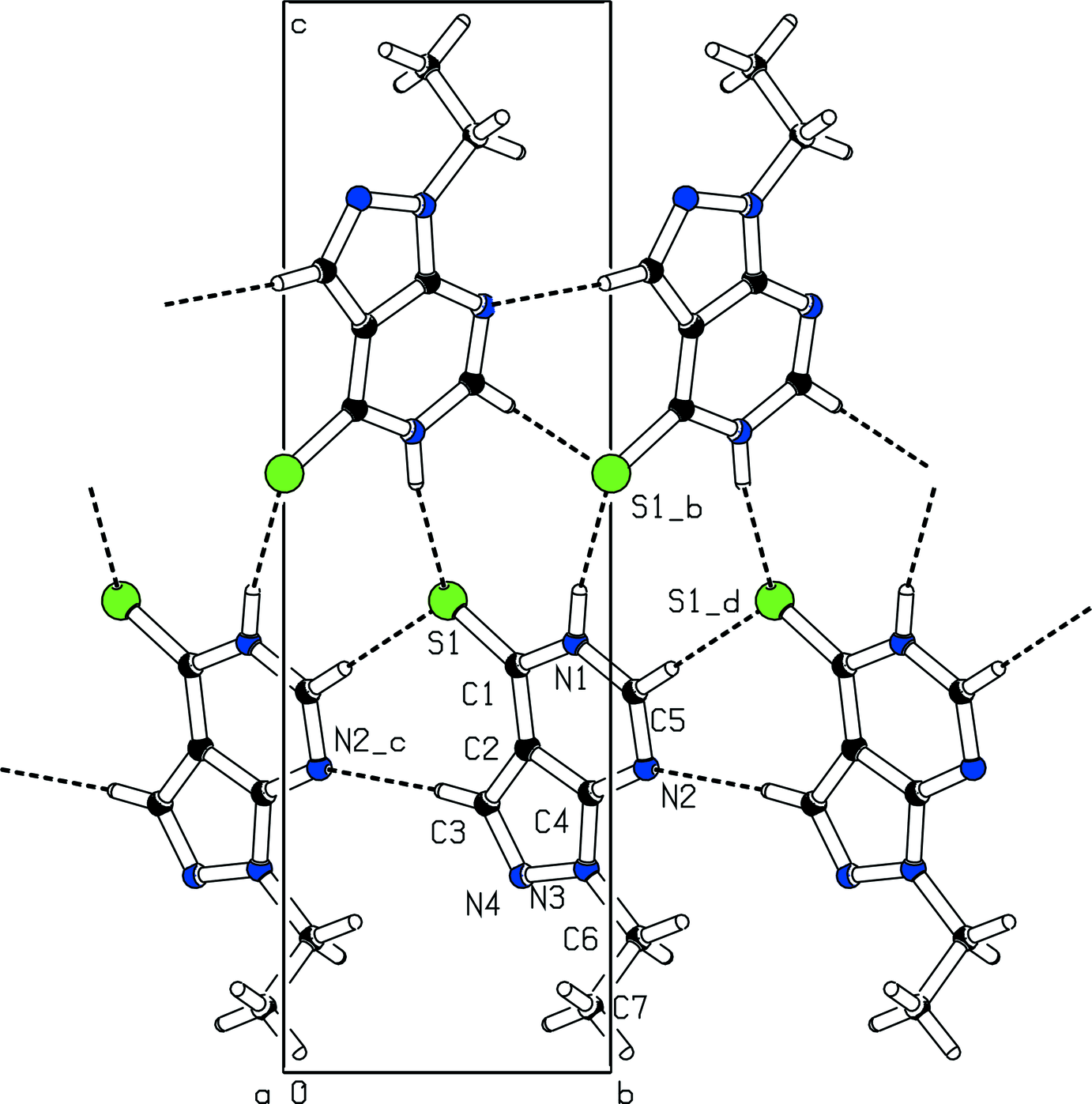

Supplement: Supplementary file 5 [file e-70-o1005-fig2.tif]
